# Supplementary figures and images for: Effects of supplemental feeding on the fecal bacterial communities of Rocky Mountain elk in the Greater Yellowstone Ecosystem
Source: PLoS One. 2021 Apr 8;16(4):e0249521. doi: 10.1371/journal.pone.0249521 (PMC8031386; doi:10.1371/journal.pone.0249521)

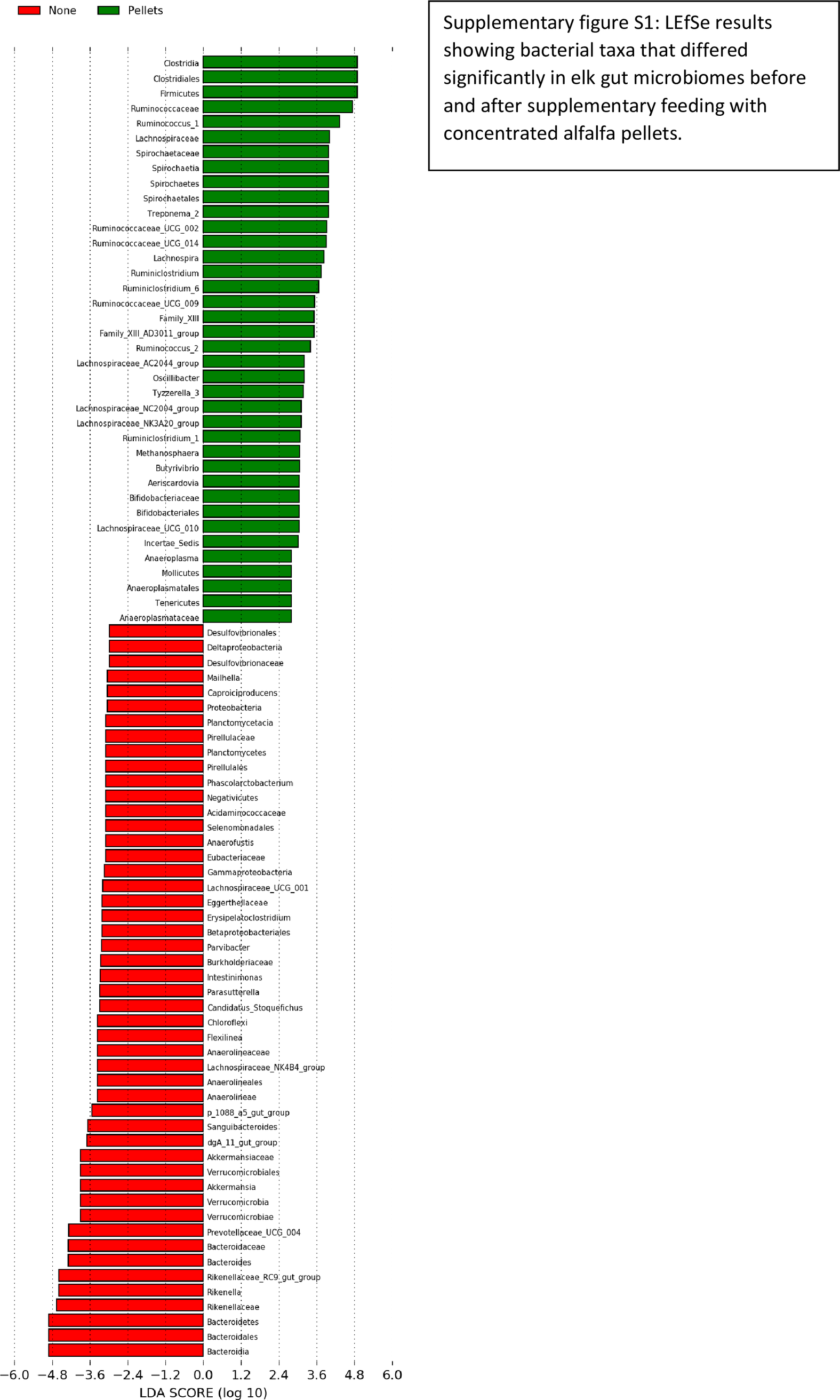

Supplement: S1 Fig — (TIF) [file pone.0249521.s001.tif]
