# Supplementary material for: Effects of supplemental feeding on the fecal bacterial communities of Rocky Mountain elk in the Greater Yellowstone Ecosystem
Source: PLoS One. 2021 Apr 8;16(4):e0249521. doi: 10.1371/journal.pone.0249521 (PMC8031386; doi:10.1371/journal.pone.0249521)
Supplement: S1 Table — (DOCX) [file pone.0249521.s002.docx]

| Location | Date | Crude Protein % | Digestible Protein % | Crude Fat % | Fiber % | NFE % | Total Carb % | Ash % | Digestible Nutrients % | Nitrogen % | Sulfur % | Phosphorus % | Potassium % | Magnesium % | Calcium % | Sodium % | Iron ppm | Aluminum ppm | Manganese ppm | Copper ppm | Zinc ppm |
| --- | --- | --- | --- | --- | --- | --- | --- | --- | --- | --- | --- | --- | --- | --- | --- | --- | --- | --- | --- | --- | --- |
| Muddy Creek | 1/22/2019 | 13.13 | 9.45 | 1.7 | 42.35 | 36.45 | 78.8 | 6.38 | 58.4 | 2.1 | 0.13 | 0.02 | 1.06 | 0.27 | 1.47 | 0.18 | 81 | 16 | 24 | 12 | 16 |
| National Elk Refuge | 2/24/2019 | 16.69 | 12.02 | 1.12 | 31.8 | 34.09 | 65.89 | 16.3 | 53.45 | 2.67 | 0.23 | 0.22 | 1.9 | 0.31 | 2.01 | 0.18 | 631 | 324 | 61 | 9 | 22 |
| Forest Park | 1/19/2019 | 7.31 | 3.66 | 1.04 | 38.58 | 43.09 | 81.67 | 9.98 | 49.26 | 1.17 | 0.12 | 0.15 | 1.78 | 0.22 | 0.68 | 0.04 | 180 | 51 | 72 | 6 | 17 |
| Alpine | 1/19/2019 | 12.81 | 6.41 | 1.49 | 40.02 | 38.28 | 78.3 | 7.4 | 50.53 | 2.05 | 0.1 | 0.3 | 2.09 | 0.16 | 1.15 | 0.03 | 92 | 24 | 24 | 7 | 21 |
| Fall Creek | 1/24/2019 | 15.56 | 11.21 | 1.08 | 37.66 | 36.78 | 74.44 | 8.92 | 57.57 | 2.49 | 0.2 | 0.21 | 2.71 | 0.25 | 1.27 | 0.08 | 95 | 30 | 40 | 9 | 17 |
| Dell Creek | 1/24/2019 | 8.25 | 4.13 | 1.07 | 40.08 | 44.9 | 84.98 | 5.7 | 51.56 | 1.32 | 0.1 | 0.18 | 1.63 | 0.17 | 0.72 | 0.04 | 70 | 15 | 30 | 7 | 13 |
| Soda Lake | 1/25/2019 | 6.69 | 3.34 | 1.05 | 38.92 | 42.45 | 81.37 | 10.89 | 48.8 | 1.07 | 0.22 | 0.13 | 2.09 | 0.16 | 0.5 | 0.02 | 73 | 20 | 81 | 5 | 14 |
| Black Butte | 1/22/2019 | 10 | 5 | 1.46 | 32 | 50.25 | 82.25 | 6.29 | 51.08 | 1.6 | 0.17 | 0.18 | 1.71 | 0.16 | 0.4 | 0.02 | 102 | 12 | 91 | 7 | 26 |
| Green River | 1/23/2019 | 7.88 | 3.94 | 1.05 | 35 | 50.96 | 85.96 | 5.12 | 51.79 | 1.26 | 0.12 | 0.15 | 1.49 | 0.15 | 0.41 | 0.04 | 169 | 17 | 129 | 5 | 18 |
| Fish Creek | 1/20/2019 | 10.94 | 5.47 | 1 | 40.22 | 40.94 | 81.16 | 6.9 | 50.8 | 1.75 | 0.12 | 0.18 | 1.12 | 0.19 | 1.12 | 0.05 | 121 | 59 | 65 | 7 | 16 |
| South Park | 4/4/2019 | 10 | 5 | 1.38 | 39.92 | 41.35 | 81.27 | 7.35 | 50.65 | 1.6 | 0.08 | 0.17 | 2.41 | 0.11 | 0.71 | 0.02 | 79 | 29 | 26 | 5 | 14 |
| South Park | 1/22/2019 | 13.13 | 6.56 | 1.32 | 39.48 | 38.52 | 78 | 7.56 | 50.39 | 2.1 | 0.13 | 0.24 | 2.31 | 0.12 | 0.31 | 0.01 | 73 | 8 | 37 | 8 | 18 |
| South Park | 3/11/2019 | 16.81 | 8.41 | 1.19 | 30.97 | 36.65 | 67.62 | 14.38 | 46.37 | 2.69 | 0.17 | 0.28 | 1.65 | 0.45 | 2.3 | 0.03 | 1025 | 390 | 141 | 8 | 30 |
| Horse Creek | 1/22/2019 | 17.19 | 8.59 | 1.82 | 34.53 | 37.88 | 72.41 | 8.58 | 49.66 | 2.75 | 0.2 | 0.32 | 2.68 | 0.17 | 0.64 | 0.01 | 550 | 128 | 77 | 7 | 35 |
| Horse Creek | 4/4/2019 | 17.69 | 8.84 | 1.02 | 32.5 | 39.27 | 71.77 | 9.52 | 48.96 | 2.83 | 0.16 | 0.29 | 1.87 | 0.3 | 1.47 | 0.03 | 185 | 106 | 42 | 8 | 28 |
| Horse Creek | 3/11/2019 | 9.13 | 4.56 | 1.33 | 38.76 | 42.63 | 81.39 | 8.16 | 50.22 | 1.46 | 0.13 | 0.22 | 2.32 | 0.13 | 0.48 | 0.01 | 107 | 42 | 42 | 5 | 21 |

Table S1: Nutrient content analysis results from concentrated feed (National Elk Refuge only) and hay (all other locations) at a subset of sites and time points over the course of this study.
