# Supplementary material for: Effects of supplemental feeding on the fecal bacterial communities of Rocky Mountain elk in the Greater Yellowstone Ecosystem
Source: PLoS One. 2021 Apr 8;16(4):e0249521. doi: 10.1371/journal.pone.0249521 (PMC8031386; doi:10.1371/journal.pone.0249521)
Supplement: S2 Table — (DOCX) [file pone.0249521.s003.docx]

| Table S2. Modified oligonucleotides used in multiplex quantitative (q)PCR assays for F. nechrophorum ssp. nechrophorum, ssp. funduliforme, and cervid DNA and their melting temperatures. Bold and underlined nucleotides indicate locked nucleic acids. | | | |
| --- | --- | --- | --- |
| Oligonucleotide | Sequence (5' to 3') | Tm (°C) | Original Publication |
| *gyrB-forward* | **A**GG**AT**TGCATGGAGTAGGA | 63.0 | Jensen et al. 2007 |
| *gyrB-reverse* | CC**TA**TTTCATTTCGACAATCCA | 62.9 | Jensen et al. 2007 |
| *nechrophorum-specific probe* | Cy3 - TC**TA**CTTTGGAGGTTGGAGAAACAA | 67.6 | Jensen et al. 2007 |
| *funduliforme-specific probe* | Cy5 - TCCGCTTTAGAGGCTGGAGAAACG | 67.7 | Jensen et al. 2007 |
| *cervid3b-forward* | CAATAAGGCACAAACATTGACATGT | 63.0 | Keltenbrunner et al. 2018 |
| *cervid3b-reverse* | CTTGTCAATGATGATGAGGTAAGCA | 63.2 | Keltenbrunner et al. 2018 |
| *cervid3b probe* | 6FAM - T**TC**TGA**T**ATCATACACTTAAGCCAA | 66.9 | Keltenbrunner et al. 2018 |
