# Supplementary material for: Effects of supplemental feeding on the fecal bacterial communities of Rocky Mountain elk in the Greater Yellowstone Ecosystem
Source: PLoS One. 2021 Apr 8;16(4):e0249521. doi: 10.1371/journal.pone.0249521 (PMC8031386; doi:10.1371/journal.pone.0249521)
Supplement: S1 Appendix — (DOCX) [file pone.0249521.s004.docx]

Design and optimization of *Fusobacterium necrophorum* qPCR assay

For each sample, DNA was extracted from 200 mg of dry fecal matter using the procedure referenced in the main manuscript. Genomic DNA was stored at -20 C until processing and shipped on dry ice to the facility where qPCR was conducted. In addition to measuring the dry mass of fecal samples for cross-sample normalization of *F. nechrophorum* DNA quantities, a multiplex quantitative (q)PCR assay was developed which quantifies both parasite and host DNA. With this approach absolute quantities of *F. nechrophorum* DNA can be normalized to the amount of cervid DNA in the same sample. Normalizing parasite DNA quantities to host DNA quantities allows approximate parasite load to be compared across samples. The cervid assay also served as an exogenous internal positive control (IPC) to gauge amplification success. To enumerate and normalize the amount of fusobacterium DNA in fecal samples we combined the sub-species-specific fusobacteria assays of Jenson et al. 2017 with an assay designed by Kaltenbrunner et al. 2018 that broadly amplifies corvid species, including rocky mountain elk. Modifications were made to each assay to reduce bias and improve sensitivity and specificity in multiplex quantitative (q)PCR reactions. The sequences of the *gryB-forward*, *gyrB-reverse*, and *necrophorum-specific* probe were identical to those of the original sequences except that locked nucleic acids (LNAs) were used in several positions in each sequence (see supplemental Table 1). The *funduliforme-specific* probe sequence was modified slightly (5’ “AC” nucleotides removed) to equalize the melting temperatures of the two sub-species-specific probes. Assay “3b” was selected from Kaltenbrunner et al. 2018 because the amplicon size and the melting temperatures were most similar to the *F. necrophorum* assays. However the probe was modified by substituting in three LNAs at the 3’ end and adding “ACC” to the 5’ end to raise the melting temperature to that of the *F. necrophorum* assay probes. Each probe was fluorescently quenched by 3’-IowaBlack quencher (idtdna.com) fluorescently labelled at the 5’ end as follows: Cy3-labelled *nechrophorum-specific* probe, Cy5-labelled *funduliforme-specific* probe, and 6FAM-labelled *cervid3b* probe. All oligonucleotides were validated in-silico with the oligo-analyzer tool available from idtdna.com and with NCBI primer blast (Ye et al. 2012) to ensure that there was no cross-reactivity with non-target organisms or with oligonucleotides used in the multiplex reaction. The multiplex reaction recipe was optimized prior to testing samples using cultured *F. necrophorum* DNA extracts (acquired from DSMZ, Braunschweig, Germany) and synthetic DNA (G-Blocks synthetic gene fragments ordered from idtdna.com). Optimization involved altering relative concentrations of each oligonucleotide such that observed quantification (C_q_) values for each assay were most similar to each other across target DNA concentrations ranging from 10 to 1e^5^ gene copies per reaction.

Optimized multiplex reactions for qPCR were made according to the following recipe: 4 µL of 5x QuantaBio PerfeCTa Multiplex qPCR ToughMix, 0.4 µM of *gyrB-forward* and *gyrB-reverse* primers, 0.2 µM of the forward and reverse cervid assay primers, 0.1 µM of each of three fluorescently labeled probes, and molecular grade water to 20 µL total volume. The following thermal cycle was carried out for each sample on a BioRad CFX-96: one cycle of 95 °C for three minutes followed by 40 cycles of 95 °C for 15 seconds and annealing at 60 °C for one minute (fluorescence for each cycle was recorded at the end of the annealing phase). Each sample was amplified in triplicate in 96-well optical plates (USA Scientific catalog #1402-9500) which were sealed by hand with optical adhesive film (Applied Biosystems catalog #4360954). Each plate included a tissue positive control, a no template control (NTC), and a standard dilution of synthetic DNA fragments containing each target sequence in equal proportions. Reagent preparation was carried out in a DNA-free workspace and samples were dispensed into plates in a low-template workspace that had not previously processed cervid tissue. Analysis of amplification curves, including amplification identification, normalization, and C_q_ assignment was carried out with custom R scripts (v3.6.1, R Core Team 2017) using the following packages: chipPCR (Roediger et al. 2014), drc (Ritz et al. 2015), qpcR (Ritz et al. 2008).

Neither subspecies of *F. necrophorum* amplified in any fecal DNA extract, save for a single sample which amplified a low amount of *F. necrophorum funduliforme* DNA in all three technical replicates. All culture positive controls and synthetic gene standards amplified while none of the NTCs amplified. There were several instances where one or more of the technical replicates failed to amplify cervid DNA, indicating either that DNA was not adequately extracted from the sample or that the amplification reaction failed. Given the infrequency of positive detections of *F. nechrophorum* DNA among all samples, we elected not to rerun samples that failed IPC tests.

References

Jensen, A., Kristensen, L.H. and Prag, J., 2007. Detection of Fusobacterium necrophorum subsp. funduliforme in tonsillitis in young adults by real-time PCR. Clinical microbiology and infection, 13(7), pp.695-701.

Kaltenbrunner, M., Hochegger, R. and Cichna-Markl, M., 2018. Red deer (Cervus elaphus)-specific real-time PCR assay for the detection of food adulteration. Food Control, 89, pp.157-166.

R Core Team (2017). R: A language and environment for statistical computing. R Foundation for Statistical Computing, Vienna, Austria. URL https://www.R-project.org/.

Ritz C, Spiess A (2008). qpcR: an R package for sigmoidal model selection in quantitative real-time polymerase chain reaction analysis. Bioinformatics, 24:13(1549–1551). https://doi.org/10.1093/bioinformatics/btn227

Ritz, C., Baty, F., Streibig, J. C., Gerhard, D. (2015) Dose-Response Analysis Using R PLOS ONE, 10(12), e0146021

Roediger S and Burdukiewicz M (2014). chipPCR: Toolkit of helper functions to pre-process amplification data. R package version 0.0.8-4

Ye J, Coulouris G, Zaretskaya I, Cutcutache I, Rozen S, Madden T (2012). Primer-BLAST: A tool to design target-specific primers for polymerase chain reaction. BMC Bioinformatics. 13:134.
