# Supplementary material for: Effects of supplemental feeding on the fecal bacterial communities of Rocky Mountain elk in the Greater Yellowstone Ecosystem
Source: PLoS One. 2021 Apr 8;16(4):e0249521. doi: 10.1371/journal.pone.0249521 (PMC8031386; doi:10.1371/journal.pone.0249521)
Supplement: Attachment — Submitted filename: Reviewer_response.docx [file pone.0249521.s005.docx]

Response to reviewers

*We are grateful to the editor and reviewers for their thorough and insightful comments and have addressed their concerns to the best of our abilities. Each of the editor/reviewer comments is listed below in red, with our responses in black italicized text. Please note that, in addition to the changes suggested by the editor and reviewer, we have also implemented several minor changes suggested during an internal USGS review process.*

Editor:

1. Please ensure that your manuscript meets PLOS ONE's style requirements, including those for file naming.
2. In your Methods section, please provide additional information regarding the permits you obtained for the work. Please ensure you have included the full name of the authority that approved the field site access and, if no permits were required, a brief statement explaining why.

*We have added an explanation to the methods section at lines 146-149 explaining why project-specific permits were not required.*

3. We note that you have stated that you will provide repository information for your data at acceptance. Should your manuscript be accepted for publication, we will hold it until you provide the relevant accession numbers or DOIs necessary to access your data. If you wish to make changes to your Data Availability statement, please describe these changes in your cover letter and we will update your Data Availability statement to reflect the information you provide.

*We still intend to provide repository information prior to publication.*

4. We note that Figure1 in your submission contain map images which may be copyrighted. All PLOS content is published under the Creative Commons Attribution License (CC BY 4.0), which means that the manuscript, images, and Supporting Information files will be freely available online, and any third party is permitted to access, download, copy, distribute, and use these materials in any way, even commercially, with proper attribution. For these reasons, we cannot publish previously copyrighted maps or satellite images created using proprietary data, such as Google software (Google Maps, Street View, and Earth). For more information, see our copyright guidelines: <http://journals.plos.org/plosone/s/licenses-and-copyright>.

*The DEM and river/hydrology layers are USGS "owned" and the feedground locations are public information, so to the best of our knowledge, the whole figure is not copywrite-able and is in the public domain. The creator of the figure, Kimberly Szcodronski, is an author on the paper. We have added the text “map courtesy of the U.S. Geological Survey” to the end of the figure caption for clarity.*

5. Please include captions for your Supporting Information files at the end of your manuscript, and update any in-text citations to match accordingly.

*We have added the required captions and updated in-text citations to match.*

Reviewer #1:

The manuscript entitled Effects of supplemental feeding on the gut microbiomes of Rocky Mountain elk in the Greater Yellowstone Ecosystem by Couch and colleagues attempts to describe the influence of supplemental feeding on the gastrointestinal microbiota of Rocky mountain Elk in the greater Yellowstone ecosystem, specifically at several feeding grounds in Wyoming. The paper is well written, samples appear to have been appropriately processed and analyses are mostly appropriate but are let down by several frail assumptions that negate the conclusions of the paper.

First and foremost is that samples are ground-collected fecal pellets. While I am keenly aware of the challenges of sampling wild animals, including Elk, it should be noted that microbiota detected in fecal pellets are significantly different from those detected in the rumen (e.g. Perea et al. DOI: 10.2527/jas.2016.1222), the paper I have provided as an example here may be important to reconciling this first issue as it not only shows these differences explicitly but also points to the importance of the distal gut (incl. fecal) microbiota to nutrition, measured therein as Feed efficiency. That being said, the discussion, including in the abstract on ruminal acidosis is not only speculative it is irrelevant to the study design.

*We understand the reviewer’s concerns regarding the biospatial delineations among GI tract microbiota, and we address this issue specifically regarding elk on line 347-350. However, the study we reference for ruminal acidosis-associated changes (line 339-340, Plaizier et al. 2017) reveals changes to the fecal microbiome in addition to the rumen microbiome, several of which are also observed in our study. Therefore, we regard the potential connection between the fecal microbiome and rumen acidosis in elk to be worthy of discussion and relevant to future research.*

*Moreover, although Perea et al. identifies biospatial differences in the GI tract microbiota of lambs, the study also identifies feed efficiency-associated shifts that are consistent between the rumen and fecal microbiomes (e.g. OTU 3 was found to be more abundant in both the rumen and the feces of efficient vs inefficient lambs). Therefore, it is conceivable while sites along the GI tract host distinct microbial communities, individual taxa may exhibit trends that are consistent between GI tract regions.*

Next, the collection of pellets from the ground will certainly carryover soil and other environmental microbes, thus conclusions around 'low-abundance bacterial genera' can not conclusively be attributed to differences in gut microbial populations.

*Pellets were collected from snow-covered ground, which, while not sterile, likely precluded most soil microbes. We have added this information at line 130. Previous studies of snow surface microbiomes indicate extremely low biomass (<1,000 reads/ml snowmelt) and unique dominant taxa that were not present among the low-abundance taxa we discuss here (e.g. Michaud et al. 2014, DOI: 10.1371/journal.pone.0104505).*

Finally, there is no knowledge of how many elk are represented by the pellets collected - they could theoretically represent a single animal at each sampling site, in which case the differences could simply reflect inter-individual variation.

*This is an excellent point, and we recognize that we did not give sufficient information to address this concern in the original manuscript. Although possible, it is extremely unlikely that duplicate samples were collected from the same individual at any given time point, because we collected very fresh samples that were still warm and not frozen, and outside temperatures generally far below freezing at time of collection. Given that the defecation rate of elk is approximately once per hour when elk are awake and active (Clinton Epps, personal communication), we believe we may safely assume that samples were not repeated within individuals at each time point. We have added a more thorough explanation of our methods and assumptions at lines 130-134.*

Secondly, the sample sites are all within 8 - 12 miles of one another; given Elk can move this distance in a day, and that diet-induced transitions of the gut microbiota can take 3-4 weeks, how can the authors be certain the samples are reflective of elk strictly consuming the supplemental feeds at each feed station and not elk that have recently moved to one or other feed station?

*This is another valid concern that was not addressed sufficiently in the original manuscript. GPS collar data demonstrates that the vast majority of elk remain at a single location all winter, rarely moving more than 5 km away (see Appendix 2 of Merkle et al. 2017). We are therefore confident that samples reflect the diets being fed at each feedground. We have improved our explanation of movement at lines 113-115.*

Other concerns:

Sampling depth appears excellent, but coverage is not reported

*Based on the context, it is unclear whether the reviewer is referring to “coverage” in terms of redundancy per base (averaged across bacterial sequence variants), or total number of usable reads from the sequencing machine. We have added the latter value to the manuscript at line 173, as it is likely to be of more general interest and can be used to estimate the former value if desired.*

The authors report differences in richness, but oddly do not look at alpha diversity, which is one of the most reliable measures of dysbiosis.

*Species richness is itself a measure of alpha diversity, and arguably more transparent and easier to interpret than indices that incorporate evenness. Preliminary analyses demonstrated that Shannon diversity exhibited very similar patterns to observed richness. However, we opted to include only richness as we were primarily interested in beta diversity and composition, and including additional metrics of alpha diversity did not assist in addressing our hypotheses.*

The F. necrophorum qPCR data is oddly tacked on, shows nothing of consequence and should be removed.

*We agree that the qPCR results appear somewhat tangential to the overall findings of the paper, however, the original intent of the study was to identify correlations between F. necrophorum abundance and microbiome community variation, and it was with this intent that we developed and validated this qPCR assay. The protocol we developed is unlikely to be published if not included in this paper, and we believe its publication will be valuable to other researchers studying F. necrophorum in elk. Given that PLOS One evaluates research on the basis of scientific validity rather than impact, we hope that we will be permitted to include the F. necrophorum protocol and results in this publication.*
