# Supplementary material for: Effects of supplemental feeding on the fecal bacterial communities of Rocky Mountain elk in the Greater Yellowstone Ecosystem
Source: PLoS One. 2021 Apr 8;16(4):e0249521. doi: 10.1371/journal.pone.0249521 (PMC8031386; doi:10.1371/journal.pone.0249521)
Supplement: Attachment — Submitted filename: Response to Academic Editor and Reviewer Nov 2020.docx [file pone.0249521.s006.docx]

Response to Academic Editor and Reviewer:

*Please see below for point-by-point responses to the editor and reviewer comments accompanying the rejection of our manuscript. Editor and reviewer comments are copied and pasted exactly as they were written, and our responses are shown in italicized red font.*

*The below text is copied directly (including capitalization and punctuation) from the rejection email we received from Dr. Juan J. Loor, the Academic Editor who handled this manuscript:*

Dear Dr. Couch,

Thank you for submitting your manuscript to PLOS ONE. After careful consideration, we have decided that your manuscript does not meet our criteria for publication and must therefore be rejected.

Specifically:

MAJOR ISSUES RAISED IN THE ORIGINAL REVIEW REMAIN AS IT SEEMS THAT AUTHORS DID NOT SERIOUSLY CONSIDER THEM.

I am sorry that we cannot be more positive on this occasion, but hope that you appreciate the reasons for this decision.

Yours sincerely,

Juan J Loor
Academic Editor
PLOS ONE

*Our response to this comment:*

*While we appreciate the time and effort the Academic Editor devoted to evaluating this manuscript, their specific reasons for rejection are unclear based on the limited justification provided. We are concerned that, because both the first and second rounds of reviews were conducted by a single reviewer (apparently the same individual in both instances), the editor’s decision was based on insufficient information. Additionally, we do not agree with the editor’s statement that we did not take reviewer concerns seriously. While we respectfully disagreed with a small number of the reviewer’s initial comments, we considered their arguments very seriously and attempted to thoroughly address and explain the logic underlying our disagreements (please refer to our initial reviewer response). In the second round of review, the primary concern of the reviewer seems to be our difference of opinion regarding the inclusion of methodology and null results from an assay we developed and validated as part of this study. To our understanding, a primary goal of PLOS ONE is to evaluate research based on scientific merit rather than impact or significance of the results. We therefore request that the decision to reject this manuscript, which appears to conflict with the mission of PLOS ONE, be reconsidered.*

*The reviewer’s only comment from this round of review is shown below:*

Rather than addressing several significant concerns, the authors have elected to be dismissive leaving a manuscript that lacks rigor with conclusions built on multiple unprovable assumptions given the data provided and study design. Collectively it is hard to see what value it adds to the literature. This is perhaps best exemplified by the persistent inclusion of F. necrophorum qPCR data that, by the authors own admission are irrelevant because it is unlikely to be published elsewhere. For future it should also be noted that diversity by definition is a measure of both richness and evenness, therefore richness is not a complete measure of alpha-diversity, further alpha-diversity, including evenness is a more faithful indicator of dysbiosis which was the point the author is trying to speak to.

*Our response to this comment:*

*The reviewer makes three main points: (1) we were overly dismissive of the reviewer’s concerns, (2) our manuscript lacks rigor with conclusions based on unprovable assumptions, (3) we included methodology and null findings from a qPCR assay developed as part of this study, and (4) we opted to use observed richness as a measure of alpha diversity. We will address each point below:*

1. *Perhaps the validity of this concern can be best evaluated by referring our original response to the reviewer (attached). While we respectfully disagreed with the reviewer on several points, we took their comments very seriously and attempted to provide full, transparent, and logical justification for these disagreements. We request that the individual/s reviewing this resubmission refer to our original reviewer response to evaluate the validity of this concern.*
2. *The reviewer does not provide specific instances of unprovable assumptions or lack of rigor. The specific concerns raised by the reviewer (points 3 & 4) do not relate to rigor or assumptions, but rather differences of opinion regarding the value of including null results and the relative merit of different measures of alpha diversity. However, as our original responses to points 3&4 were unsatisfactory to the reviewer, we have attempted to revise our manuscript to better address their concerns (see below for specifics).*
3. *As explained in our original rebuttal letter, we wish to retain methodology and null findings from our F. necrophorum qPCR assay because we believe its publication will be valuable to other researchers studying this pathogen. Given that PLOS ONE evaluates research on the basis of scientific validity rather than perceived impact, we hope to include the methodology and findings in this manuscript.*

*We have attempted to better integrate the F. necrophorum methods, results, and significance more cohesively into the manuscript by revising/adding explanation of methods at lines 166-168 and results at lines 227-230. The importance of our findings are discussed at lines 357-365. However, if a second reviewer also recommends that we remove the qPCR material, we would be happy to reconsider.*

4. *The reviewer objects strongly to our use of observed richness as a measure of alpha diversity rather than an index that incorporates evenness. According to the reviewer, alpha diversity by definition includes a measure of evenness. However, Whittaker’s original definition of alpha diversity specifically states that it is a measure of the number of species in a locality or habitat (Whittaker 1960, Ecological Monographs), not to be confused with Fisher’s alpha, which is a diversity index. In microbiome research, observed richness normalized by sampling depth is a commonly used measure of alpha diversity. Evenness estimates from amplicon sequence data can be skewed by sequencing bias and data aggregation, and diversity indices that include evenness are more difficult to interpret than observed richness. Additionally, the reviewer states that evenness is one of the most reliable indicators of dysbiosis, but they fail to present any references to support this claim. To our understanding, this argument does not align with the literature. In many studies of the gut microbiome, reduction in species richness is a primary indicator of dysbiosis, though the reviewer is correct that this pattern frequently coincides with reduction in evenness and other alpha diversity indices. We therefore respectfully disagreed with the reviewer’s recommendation during the first round of reviews.*

*However, as our justification for omitting alpha diversity metrics was unsatisfactory to the reviewer, we have now added Inverse Simpson and Shannon diversity comparisons to the cross-sectional analysis at lines 182-193 and in Figure 2. Results from this analysis are consistent with the previously reported observed richness results (lines 223-227, Figure 2). Therefore, inclusion of the alpha diversity indices did not alter our conclusions.*
