# Supplementary material for: Effects of supplemental feeding on the fecal bacterial communities of Rocky Mountain elk in the Greater Yellowstone Ecosystem
Source: PLoS One. 2021 Apr 8;16(4):e0249521. doi: 10.1371/journal.pone.0249521 (PMC8031386; doi:10.1371/journal.pone.0249521)
Supplement: Attachment — Submitted filename: Response_to_reviewers.docx [file pone.0249521.s007.docx]

**Editor and Reviewer Responses**

*Editor and reviewer comments are listed in nonitalicized black font. Author responses are shown in red italics.*

*Editor comments:*

With regards to diversity metrics, I tend to agree with the reviewers and feel that the way that most metrics are taught, presented, and interpreted make them seem more interchangable than they are. The authors likely know all this, and I include the explanation here not to pander but to explain my view, which I believe is shared by multiple reviewers.  To the point about evenness, this information is often incorporated into calculations such as Shannon, or Simpsons, in some way, but I find that it is possible to obscure trends in either richness or evenness by combining them into a holistic diversity metric.  I used to use Shannon's all the time, and now I find it is more informative to use richness and evenness specifically, because they reveal more important trends.  For example, whether all bacteria are equally affected by a treatment which reduces richness, or only certain members. Thus, the authors have previously tried to address the concern over diversity metrics by adding additional ones, but I think this point can best be settled by the authors verifying that the diversity metrics they have selected indeed provide the information they find most pertinent to their study.

*We have attempted to clarify why the diversity metrics we have selected provide the most pertinent information to our study by adding the following text at lines 183-184 explaining our choice of indices: “Inverse Simpson and Shannon diversity indices, both of which incorporate taxonomic evenness in addition to richness, were also calculated in phyloseq [47] to assess whether alpha diversity results were especially sensitive to changes in rare taxa (Shannon) or common taxa (Inverse Simpson).”*

With regards to the qPCR data for testing one particular bacteria, the reviewers had mixed opinions on its usefulness, but I think this can best be addressed by adding a few extra sentences of justification for why this particular bacteria was selected, when there are many potential pathogens the authors could have chosen.  It seems like the authors chose this species because it is of concern in feedlot sheep, and may also be of concern in feedlot-raised elk, as well.  If this is the case, the authors should more explicitly state this reasoning.

*The rationale for selecting Fusobacterium necrophorum was because it is a pathogen of concern for Wyoming elk. We have clarified this at line 118.*

Reviewer #2: This study characterized the changes in the gut microbiomes of wild elk under different supplemental feeding regimes and provides useful information on how alfalfa pellets supplemental feeding can alter the native gut microbiome of wild animals and impact their health. The authors carried out comprehensive analyses of the microbiome data.

For the two major disputes from the previous review. The following are my comments/recommendations.

1. In my opinion, the authors have adequately addressed the alpha-diversity query. There is some terminology confusion between “diversity” and “alpha-diversity”, particularly in how they are used in macro and microbial ecology, but I don’t think it’s fair to overly penalize the authors for such a minor terminology difference that is widespread in the field.

I do think it’s always a good idea to look at multiple alpha-diversity metrics and it’s more convincing now that they have added in two other metrics (which both represent evenness to some degree).

The degree to which richness or evenness better capture dysbiosis is definitely a contentious point. I think the previous reviewer may actually be right regarding the importance of evenness, but that’s just my opinion and far from accepted in the field - richness is the more commonly compared metric.

In conclusion, I think what the authors provided have sufficiently address the issue.

2. For the qPCR measurement of the F. necrophorum, I think it will be okay to keep. However, the writing of this experiment in the Result is not clear. I am copying exactly what is the manuscript below starting from Line 227. “The F. necrophorum qPCR assay did not detect either strain in any of the elk fecal samples, save for a single sample that amplified a low amount of F. necrophorum funduliforme, suggesting that these species are rarely shed in elk feces”
First, the period at the end of the sentence is missing. More importantly, I don’t think this sentence is correct or clear. This sentence needs to be revised for clarify.

*We have made the following revision for clarity at lines 231-233: “F. necrophorum funduliforme was identified in only a single fecal sample, and F. necrophorum necrophorum was not detected in any samples, suggesting that these species are rarely or never shed in elk feces.”*

Other than the two points above, I am in favor of accepting this manuscript for publication as it certainly contains helpful information for the field of wild life microbiome studies.

Reviewer #3: Manuscript Number: PONE-D-20-13745R2
Manuscript Title: Effects of supplemental feeding on the gut microbiomes of Rocky Mountain elk in the Greater Yellowstone Ecosystem

The objective of this current study was to assess the impact of supplemental winter feeding on the gut microbiome of Rocky Mountain elk in western Wyoming exploring the potential implications of feeding on the elk population health and disease. This is a descriptive study of the bacterial diversity in fecal samples from a large number of wild, free-ranging elk on either natural pasture or fed supplemental feed at 14 different locations and timepoints (8-23 samples per time point, 273 samples in total according to Table 1).

Title
The title should reflect the fact that fecal samples were analyzed – not “gut” samples – as noninvasive sampling was employed without sacrificing animals and sampling the different sections of their digestive tract. This wording should be corrected throughout the manuscript to avoid misunderstandings and to be clear about the identity of the samples. The title should also communicate that only the bacterial microbiome of the feces was analyzed. The ruminant rumen and hindgut microbiome includes both bacteria, ciliates, anaerobic fungi and methanogens.

*The title has been changed to “Effects of supplemental feeding on the fecal bacterial communities of Rocky Mountain elk in the Greater Yellowstone Ecosystem”.*

Elk are intermediate ruminants
As far as I can see, the word “ruminant” is only mentioned twice in the manuscript, fist at the end of the introduction when describing the work to develop an elk-specific assay to assess the abundance of Fusobacterium necrophorum, and then one more time at the end of the discussion when talking about this widespread commensal in elk feces. The fact that the elk is a ruminant is a key feature to understand it´s gut microbiome, and the effect of diet and supplemental feeding on the gut microbiome and the health of the host animal. I miss a discussion on the physiological and anatomical adaptations of these ruminants in the manuscript. Please include a description of their digestive tract with reference to the pioneering work by Hofmann, and the symbiotic microbial digestion of their herbivorous diet in the reticulorumen and the hindgut.

*A brief description of the elk digestive system has been included at lines 347-352.*

Ruminants on natural pasture with seasonal changes in appetite / food intake and exposed to seasonal changes in diet composition and chemistry show seasonal changes in their symbiotic rumen and hindgut microflora (e.g. the high-arctic Svalbard reindeer Rangifer tarandus platyrhynchus (Orpin et al 1985 <https://pubmed.ncbi.nlm.nih.gov/4026289/> )). As underlined in the abstract of this manuscript the interplay between diet, the microbiome, and the host health highlights the need to understand the consequences of supplemental feeding on the microbiomes of free-ranging “populations”. This has been studied in e.g. the semi-domesticated intermediate ruminant reindeer (Rangifer tarandus tarandus) (see review by Sundset et al. 2015 Encyclopedia of Metagenomics <https://link.springer.com/referenceworkentry/10.1007%2F978-1-4899-7475-4_664> ). Supplemental feeding in periods when access to pasture is poor and the animals may have starved is challenging and may result in rumen malfunction e.g. rumen acidosis.

Materials, methods, data
• Age and sex of the animals sampled are not presented / known (both are known to influence the composition of the gut microbiome) but may be discussed in relation to these data.

*We have added an acknowledgement of this to the discussion at lines 365-366.*

• PCR and sequencing of the 16S V4 region was performed. Please include the primer sets used under the method section on sample sequencing.

*Primers have been included at line 164*

• According to Table 1, 273 samples were collected? It is stated in the method section that the samples were split equally between two MiSeq runds including 315 elk fecal samples and including the 199 samples used in this study. This is not clear to me. What happened to the other samples? What were they used for? And then below you state that a total of 22,620,453 reads were obtained from the 315 samples? Did you use 315 or 199 samples? Or 273 samples?

*The original number of 199 in the methods section was incorrect. The total number of samples used in this study was 282. This has been corrected in the text. The additional samples from the sequencing run were from a separate study on elk disease or did not meet the minimum sequencing depth requirement.*
• Several studies exist on the microbiome in the gastrointestinal tract of wild ruminants. It would be nice to see a comparison between this current dataset from the Rocky Mountain elk feces and e.g. colonic samples from North American moose – the largest browsing ruminant of the deer family (Ishaq & Wright, 2012, BMC Microbiology 12) or datasets generated from the feces of other ruminants (wild or domestic).

*Although we agree with the reviewer that such a comparison would be useful, it is somewhat out of the scope of our hypotheses. However, sequencing data will be made publicly available for future comparisons.*

• Also, as only feces and not samples from the digestive tract were obtained, please discuss this aspect with reference to other papers comparing the feces microbiome to that of e.g. the colonic microbiome in ruminants. What are the pros and cons for using fecal samples instead of e.g. rumen samples when investigating the potential implications of feeding on the elk population health and disease?

*We have included the following text at lines 371-375: “In domestic ruminants, the foregut microbiome has higher richness and may be more responsive to feed changes than the fecal microbiome (Lourenco et al. 2020). Therefore, while the fecal microbiomes of ruminants are easily sampled noninvasively, they represent only a subset of the complex and variable gastrointestinal tract microbiome and must be interpreted with care.”*

• Did you see any health or disease problems among the animals sampled in this current study?

*No – we did not observe any obvious signs of disease. However, because disease data was not included in this study, we cannot confidently assert that disease was absent.*

Fusobacterium necrophorum
F. necrophorum is an opportunistic pathogen found in the digestive tract of both humans and animals, known to cause necrotic conditions including liver abscesses and foot rot in ruminants, with the subspecies F. n. necrophorum (biotype A) most virulent and isolated more frequently from infections (Nagaraja et al. 2005, Veterinary anaerobes and diseases 11: 239-246). The current study presents a qPCR essay for F. necrophorum and also screened the large number of fecal samples collected from elk on both natural pasture and eating supplemental feed at different locations and different times. The qPCR essay did not detect either of the two strains in any of the samples except one with low amounts of F.n. funduliforme (biotype B). Hence, this study indicates that these populations of wild elk in the Rocky Mountains rarely shed F. necrophorum. Although, F. necrophorum has indeed previously been isolated from elk (4 isolates from footrot) by Clifton et al. (2018) Veterinary Microbiology 213: 108-113.
I agree with the authors that the findings from this current study are valuable to other researchers studying this pathogen and support their publication along with the microbiome data.

Does rumen acidosis effect the fecal microbiome? And is it likely that these animals were challenged with rumen acidosis?
Discussing their findings that Proteobacteria and Verrucomicrobia were reduced in elk fed alfalfa pellets the authors refer to the study by Plaizier et al. (2016) on the effect of a grain-based subacute ruminal acidosis challenge on the rumen digesta and feces microbiota (ref 60). Plaizier et al. concluded that also the bacterial community composition in the feces was affected by the rumen acidosis challenge (altering the lower taxonomical level), but they did not identify any bacterial taxa in the feces that could be used for accurate and non-invasive diagnosis of rumen acidosis.

*The reviewer’s comment reflects the content of the abstract of Plaizier et al (ref 60), however, the main text contains the following additional information: “In the feces, the SARA challenge did not affect the relative abundances of Firmicutes and Bacteroidetes, but it decreased those of Tenericutes, Proteobacteria, Cyanobacteria, Verrucomicrobia, and Fibrobacteres, and decreased those of Spirochaetes and Actinobacteria.” Therefore, though these taxa may not be sufficient for accurate diagnosis of rumen acidosis, we suggest that the relationships described in the current study and by Plaizier et al. may be worth exploring further as potential fecal indicators of rumen acidosis. Additionally, the Plaizier et al. study identifies a reduction in Firmicutes in the rumen but not in the feces of cattle with rumen acidosis. In our study, Firmicutes increased in the feces of alfalfa-fed elk, but we have no information regarding the rumen microbiome. However, due to the differences in host physiology and study design between our current study and Plazier et al., we believe this finding warrants further exploration.*

High intakes of rapidly digestible carbohydrates such as barley or other cereals are the primary cause of rumen acidosis in ruminants. In acute situations this may result in death. I could not access ref 61 (Hattel et al. 2007) showing that rumen acidosis is the leading cause of death among captive elk (Cervus Elaphus) in Pennsylvania – but the captive elks in this previous study may have received a different diet with a higher content of soluble carbohydrates compared to the wild Rocky Mountain elk? The chemical / nutrient analysis of the supplemental feeds given to the Rocky Mountain elk in this current study however showed that the pelleted alfalfa was low in soluble carbohydrates (and high in proteins) and would perhaps not be expected to cause rumen acidosis?

*The reviewer is correct that the pelleted alfalfa does not contain excessive amounts of soluble carbohydrates and it is high in protein. However, it is also low in fiber relative to the other feed types. Previous research has demonstrated that alfalfa pellets reduce rumen pH relative to alfalfa hay in cattle (Khafipoor et al. 2007 J Animal Sci). We agree with the reviewer that we cannot conclusively diagnose rumin acidosis, and that further research is needed to understand whether feeding concentrated alfalfa pellets induces rumen acidosis in elk. However, we also believe that findings from our study should motivate future research to clarify potential links between concentrated feed and rumen biochemistry in elk, as these questions could have important conservation and management implications. These points are summarized in the text at lines 357-368.*

*Regarding ref 61, Hattel et al. determined the cause of death for 5 out of a herd of 65 elk was rumen acidosis, and therefore rumen acidosis was among the top 5 causes of mortality.*
